# An Adrenal Crisis After Denosumab Use in a Patient With Addison's Disease: A Case Report

**DOI:** 10.7759/cureus.100118

**Published:** 2025-12-26

**Authors:** Hilal Caglar, Serdar Hira, Sabri Onur Caglar

**Affiliations:** 1 Physical Medicine and Rehabilitation, Bandırma Royal Hastanesi, Balıkesir, TUR; 2 Clinical Biochemistry, Bandırma Royal Hastanesi, Balıkesir, TUR; 3 Cardiology, Bandırma Royal Hastanesi, Balıkesir, TUR

**Keywords:** addison's disease, adrenal crisis, clinical case, denosumab, osteoporosis, primary adrenal insufficiency

## Abstract

Primary adrenal insufficiency (Addison’s disease) requires lifelong glucocorticoid and mineralocorticoid replacement, and patients are vulnerable to adrenal crisis during physiologic stress or medication-related perturbations. Denosumab, a monoclonal antibody against receptor activator of nuclear factor κB ligand (RANKL), is widely used for osteoporosis treatment and is generally well tolerated. However, to our knowledge, an adrenal crisis temporally associated with denosumab administration has not been previously documented.

A 69-year-old female patient with a 35-year history of primary adrenal insufficiency was on stable replacement therapy with 5 mg of oral prednisolone daily. Due to chronic kidney disease, denosumab was selected as an alternative antiresorptive therapy for severe postmenopausal osteoporosis. Approximately 24 hours after receiving 60 mg of subcutaneous denosumab, she developed profound fatigue, vomiting, dizziness, and hypotension. Laboratory evaluation revealed hyponatremia, elevated creatinine, and low morning cortisol with elevated adrenocorticotropic hormone (ACTH), confirming adrenal crisis. Infectious, cardiac, gastrointestinal, and pharmacokinetic triggers - including medications known to accelerate glucocorticoid metabolism - were excluded. She was successfully treated with intravenous hydrocortisone and fluid resuscitation, with full clinical recovery. To our knowledge, this is the first reported case of adrenal crisis temporally linked to denosumab use in a female patient with Addison’s disease, suggesting a possible association rather than definite causality, particularly when stress-dose glucocorticoids are not provided. Clinicians should be aware of this potential association and consider individualized risk assessment, pre-treatment glucocorticoid dose adjustments, and close monitoring in patients with adrenal insufficiency receiving denosumab.

## Introduction

Primary adrenal insufficiency, also known as Addison’s disease, is a life-threatening endocrine condition characterized by insufficient production of cortisol and, in most cases, aldosterone by the cells of the adrenal cortex [1]. Patients require lifelong, continuous glucocorticoid and mineralocorticoid replacement therapy, and any intercurrent stress, such as infection, surgery, or certain medications, can trigger an adrenal crisis if steroid doses are not adjusted appropriately [1]. Because adrenal crisis can rapidly lead to hypotension, electrolyte imbalance, shock, and even death, early recognition is essential, even for clinicians unfamiliar with endocrine emergencies. Typical manifestations include severe fatigue, nausea, vomiting, abdominal pain, hypotension, and altered mental status.

Denosumab, a human monoclonal antibody targeting receptor activator of nuclear factor κB ligand (RANKL), is widely used as an antiresorptive agent in the management of osteoporosis, treatment-induced bone loss, and cancer-related skeletal disease [2]. Its safety profile is generally favorable, with the most prominent adverse effects including musculoskeletal pain, hypocalcemia, increased infection risk, and osteonecrosis of the jaw [3]. Although denosumab is not known to directly alter glucocorticoid metabolism, its immunomodulatory effects may theoretically increase physiologic stress in susceptible individuals. To our knowledge, an adrenal crisis temporally related to denosumab administration has not been clearly reported in patients with preexisting primary adrenal insufficiency. In this report, we present a patient with Addison’s disease who developed an adrenal crisis shortly after receiving denosumab for osteoporosis.

## Case presentation

**Figure 1:**
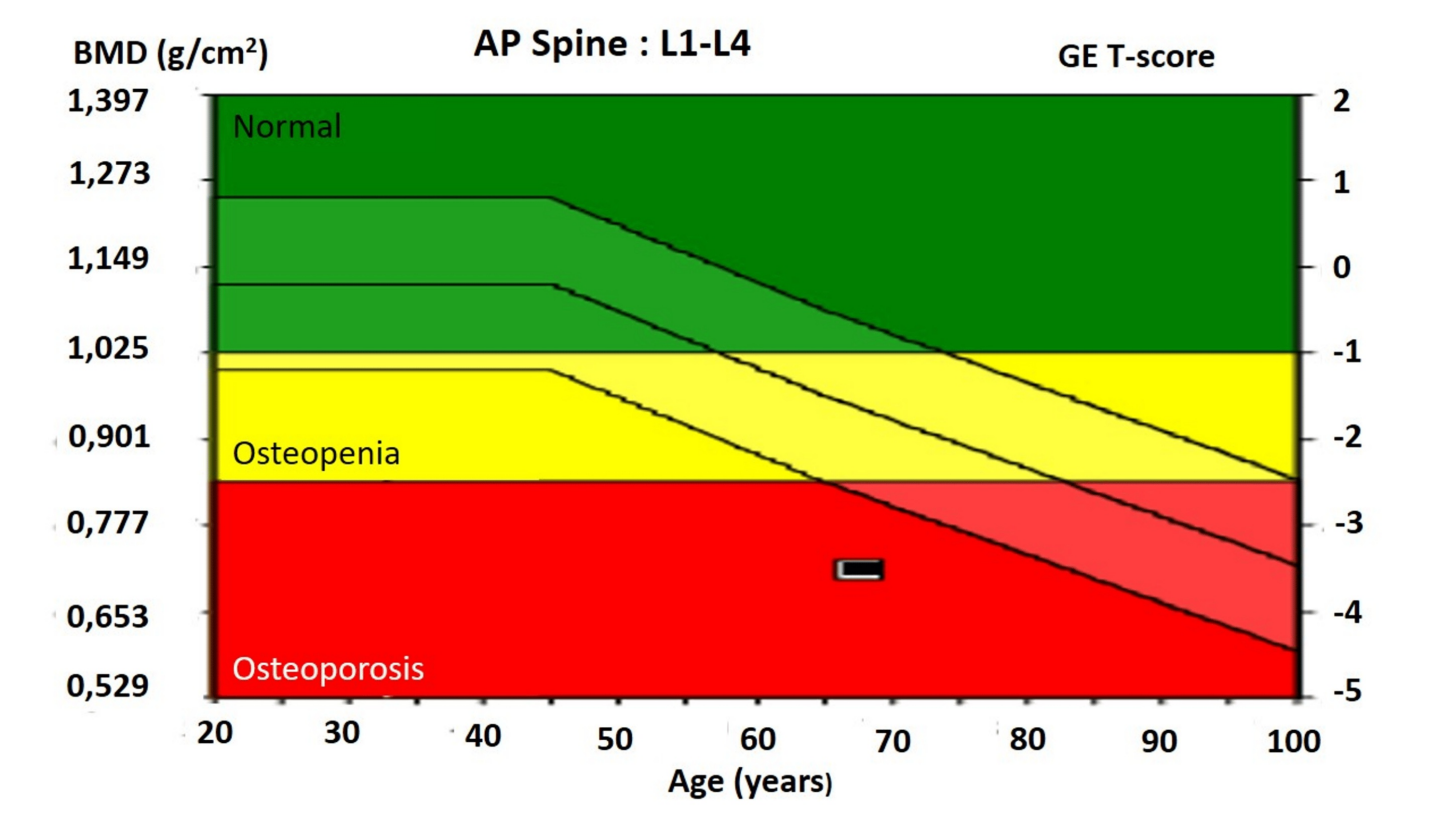
L1-L4 bone mineral density T-score reference chart illustrating the World Health Organization classification of bone density, including normal bone mass, osteopenia, and osteoporosis.

**Figure 2:**
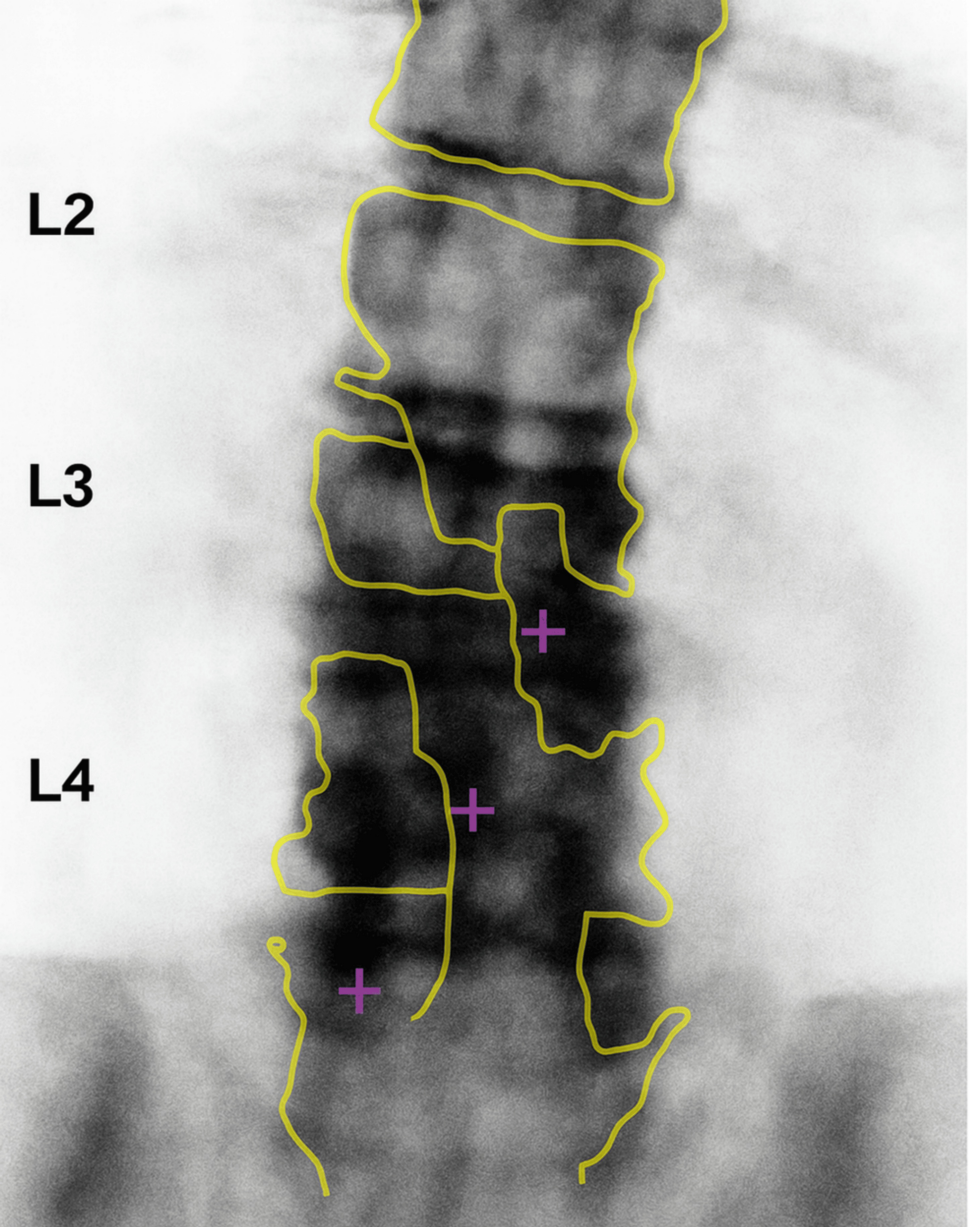
Dual-energy X-ray absorptiometry (DEXA) image of the lumbar spine (L1-L4), demonstrating reduced bone mineral density consistent with a low T-score measurement.

A 69-year-old female patient with a 35-year history of primary adrenal insufficiency due to adrenalitis was followed in the endocrinology clinic. She was on stable replacement therapy with oral prednisolone 5 mg/day, with good clinical control and no adrenal crises over the previous 25 years. Her prednisolone regimen had been unchanged for many years, and she had not required any recent dose adjustments prior to receiving denosumab. The patient was admitted to the Physical Medicine and Rehabilitation Department complaining of fatigue, myalgia, and dorsalgia. Physical examination, including neurological examination, showed no pathological findings, except for thoracic kyphosis. Her baseline laboratory evaluation was within the normal range regarding serum calcium, phosphate, alkaline phosphatase, and C-reactive protein (Table 1). Serum creatinine was 1.51 mg/dL, and glomerular filtration rate (GFR) was calculated as 32 mL/min/1.73 m² (Table 1). The patient received paracetamol 500 mg twice a day, cholecalciferol 1000 IU daily, and calcium carbonate 800 mg/day, along with physical exercise therapy. Bone density measurement was performed due to a history of chronic steroid use and suspicion of osteoporosis. Dual-energy X-ray absorptiometry (DEXA) showed postmenopausal osteoporosis with T-scores: lumbar spine -3.5, left femur neck -2.4, and left femur total -1.6 (Figures 1-2). Because of renal impairment, denosumab was selected as an alternative antiresorptive agent instead of bisphosphonates [4]. She received subcutaneous denosumab 60 mg. No stress-dose increase in her usual prednisolone therapy was made at the time of denosumab administration, as no intercurrent illness or procedural stress was anticipated.

**Table 1:** Vital parameters and laboratory values before and after denosumab administration, and at discharge, with normal ranges.

Test	Before administration	After administration	At discharge	Reference ranges
Vital parameters				
Blood pressure (mmHg)	110/60	80/50	120/70	<120, <80
SpO2 (%)	99	96	99	95-100
Pulse (bpm)	70	84	71	60-100
Temperature (°C)	36.4	37.1	36.5	36.1-37.2
Complete blood count				
Hemoglobin (g/dL)	11.5	10.9	11.1	11.6-15
Hematocrit	32.7	31.7	33.2	36-44
Leukocytes (109/L)	9.1	9.9	8.1	4.5-11
Thrombocytes (109/L9)	351	371	360	150-450
Biochemistry				
Glucose (mg/dL)	124	85	120	70-99
Sodium (mmol/L)	142	128	137	135-145
Potassium (mmol/L)	4.2	4.85	4.3	3.6-5.2
Chloride (mmol/L)	101	94.6	102	96-106
Calcium (mg/dL)	9.01	7.61	8.8	8.6-10.3
Phospate (mg/dL)	3.1	2.6	3	2.5-4.5
Magnesium (mg/dL)	1.7	1.68	1.7	1.7-2.2
Creatinine (mg/dL)	1.51	1.73	1.4	0.6-1.2
Albumin (g/dL)	4.1	3.85	4.01	3.5-5.5
Glomerular Filtration Rate (mL/min/1.73m²)	32	32	41	60-75
Alanine aminotransferase (U/L)	16.6	12.4	15.5	7-55
Aspartate aminotransferase (U/L)	28.4	20.1	22.2	10-40
Alkaline phosphatase (U/L)	74	60	75	35-104
C-reactive protein (mg/L)	<5	101	40	<5
Troponin I (ng/mL)	0.01	0.02	0.01	<0.04
Lactate dehydrogenase (U/L)	95	99	100	140-280

Approximately 24 hours after the injection, she developed progressive fatigue, nausea, vomiting, abdominal pain, dizziness, and skin darkening. On the following day, she presented to the Emergency Department with marked weakness and near-syncope. On examination, she appeared acutely ill, with blood pressure 80/50 mmHg, pulse 84 beats/min, and temperature 37.1°C. She was clinically dehydrated, with peripheral coldness and hyperpigmented skin consistent with acute adrenal insufficiency. Laboratory investigations revealed: sodium 128 mmol/L, potassium 4.85 mmol/L, glucose 85 mg/dL, creatinine 1.73 mg/dL, and calcium 7.61 mg/dL (Table 1). An 8 a.m. serum cortisol measured before steroid administration was 12 mcg/dL, inappropriately low for the severity of illness, with elevated adrenocorticotropic hormone (ACTH), confirming adrenal crisis on the background of known primary adrenal insufficiency. C-reactive protein was 101 mg/L, and white blood cell count 9.9 × 10⁹/L (Table 1). Blood and urine cultures were negative. Chest X-ray, ECG, and troponin I level showed no evidence of acute infection or cardiac event.

She was promptly treated with intravenous hydrocortisone 100 mg bolus, followed by 50 mg every six hours, and aggressive fluid resuscitation with isotonic saline and dextrose. During high-dose hydrocortisone treatment, her oral prednisolone was appropriately held and replaced with parenteral hydrocortisone, in accordance with standard adrenal crisis management. Her blood pressure and symptoms improved within 24 hours, and serum sodium and potassium gradually normalized over 72 hours. After clinical stabilization, the hydrocortisone was discontinued, and prednisolone was restarted. At the six-month follow-up, she was well and in good clinical control with 5 mg prednisolone daily.

## Discussion

Drug-induced adrenal crisis is a well-known, yet likely underreported, complication in patients with adrenal insufficiency. Classic examples include rifampicin, which induces hepatic cytochrome P450 enzymes and increases cortisol clearance. Kyriazopoulou et al. reported three patients with Addison's disease, in whom rifampicin caused profound changes in cortisol kinetics; one of them experienced an adrenal crisis shortly after starting rifampicin, despite apparently adequate glucocorticoid replacement [5]. More recently, Kuo et al. demonstrated an adrenal crisis that developed within 24 hours of the first zoledronic acid infusion in a woman with Addison's disease, likely resulting from an acute-phase reaction combined with insufficient stress-dose glucocorticoid adjustment [6]. A comparable observation was reported by AlFaifi and AlMistehi, who described two patients diagnosed with adrenal insufficiency, developing adrenal crisis shortly after receiving zoledronic acid infusion [7].

In contrast to rifampicin, denosumab is not known to directly influence glucocorticoid metabolism through cytochrome P450 induction or inhibition. However, several indirect mechanisms may plausibly explain its potential to trigger adrenal crisis in susceptible patients. Denosumab may increase the risk of infections by inhibiting the RANK pathway, which may have increased physiologic cortisol requirements beyond what a fixed replacement regimen can provide [8]. Gastrointestinal adverse effects, including nausea and vomiting, reported in some patients after denosumab administration, may have impaired the absorption of oral glucocorticoids, effectively reducing bioavailable steroid levels during a period of increased need [3,8]. Moreover, patient-related factors, such as advanced age, comorbidities, concomitant medications, and inadequate stress-dose adjustment, have all been associated with an increased risk of adrenal crisis [1]. Importantly, in our patient, the usual daily prednisolone dose (5 mg/day) was continued without a temporary stress-dose increase at the time of denosumab administration. Although no acute illness was anticipated, the absence of a temporary stress-dose increase in her glucocorticoid regimen may have contributed to reduced adrenal reserve in the setting of a physiologic stressor. The rapid onset of symptoms within 24 hours of denosumab injection, biochemical confirmation of adrenal crisis, together with the absence of alternative triggers, such as infection, vomiting preceding the crisis, or medications affecting glucocorticoid metabolism, strengthens the temporal association.

Several potential alternative etiologies were systematically excluded in this case. Infectious triggers were unlikely, given the absence of fever, normal leukocyte count, negative blood and urine cultures, and unremarkable chest radiography. The patient reported no preceding gastrointestinal illness, and vomiting began only after the onset of adrenal decompensation. There was also no history of recent surgery, trauma, psychological stress, or intercurrent illness that might independently increase cortisol requirements. Additionally, the patient was not receiving any medications known to accelerate glucocorticoid metabolism - such as rifampicin, carbamazepine, or phenytoin - excluding pharmacokinetic interference. Sepsis and cardiogenic causes of shock were ruled out clinically and biochemically. Although potassium levels were within the upper normal range, this does not preclude adrenal crisis, as early presentations and partial mineralocorticoid activity from prednisolone may blunt typical hyperkalemia. Hyponatremia was consistent with cortisol deficiency-mediated antidiuretic hormone excess.

To evaluate causality, we applied the Naranjo Adverse Drug Reaction Probability Scale, which yielded a total score of 7 - consistent with a “probable” association [9]. Although insufficient stress-dose glucocorticoid supplementation alone could theoretically precipitate adrenal crisis, the clear temporal proximity to denosumab administration, the patient's clinical course, and the exclusion of alternative triggers collectively support a “probable,” rather than coincidental, association.

## Conclusions

To the best of our knowledge, this is the first reported case of adrenal crisis temporally associated with denosumab administration in a patient with known Addison’s disease. The combination of plausible mechanistic explanations, exclusion of alternative etiologies, and supportive causality scoring suggests that denosumab may act as a clinically relevant precipitant of adrenal crisis, particularly when stress-dose glucocorticoid supplementation is not provided. This interpretation does not imply definitive causality; rather, it highlights a meaningful clinical association in a high-risk patient population. Clinicians should remain aware of this potential association, and consider individualized risk assessment, pre-treatment glucocorticoid dose adjustments, and close monitoring in patients with adrenal insufficiency receiving denosumab.
